# Bipolar disorder and peripartum mood episodes: Epidemiology and clinical correlates

**DOI:** 10.1192/j.eurpsy.2021.523

**Published:** 2021-08-13

**Authors:** E. Aragno, G. Di Salvo, G. Rosso, G. Maina

**Affiliations:** 1 Department Of Neurosciences “rita Levi Montalcini”, University of Turin, torino, Italy; 2 Department Of Neurosciences Rita Levi Montalcini, University of Turin, Turin, Italy

**Keywords:** bipolar disorder, women, reproductive cycles, peripartum episodes

## Abstract

**Introduction:**

It is known that the peripartum period is a high-risk period of recurrence in bipolar disorder (BD). However, data on correlations between reproductive life events, such as age at menarche and peripartum period, are mixed in BD.

**Objectives:**

The aims of this retrospective study are to investigate the lifetime rate of peripartum mood episodes, the clinical correlates and the relationship between age at menarche and peripartum episode in a sample of women with BD.

**Methods:**

The study focused on comparisons between women with vs. without peripartum mood episodes (n = 292). Socio-demographic and clinical characteristics between women with vs without BD peripartum episode were examined through descriptive statistics.Adjusted logistic regression analysis was run to examine the association between variables.

**Results:**

In our sample, 30% had at least one BD peripartum episode. Women with peripartum episode had significantly earlier age at menarche, earlier onset of BD and longer duration of untreated disorder compared to women without peripartum episode. After adjustment, the late menarche (>15 years) was associated with lower probability of BD episodes during the peripartum period compared to normal menarche (12-14 years).

**Conclusions:**

Peripartum mood episodes are common in BD and are correlated with early onset of BD and long duration of untreated disorder. Moreover, age at menarche may be related to the risk of peripartum mood episodes. The results deserve to be deepened in further studies.

